# ADAMTS-5 Decreases in Coronary Arteries and Plasma from Patients with Coronary Artery Disease

**DOI:** 10.1155/2019/6129748

**Published:** 2019-12-16

**Authors:** Zhen Wang, Di Ye, Jing Ye, Menglong Wang, Jianfang Liu, Huimin Jiang, Yao Xu, Jishou Zhang, Jiangbin Chen, Jun Wan

**Affiliations:** ^1^Department of Cardiology, Renmin Hospital of Wuhan University, Wuhan 430060, China; ^2^Cardiovascular Research Institute, Wuhan University, Wuhan 430060, China; ^3^Hubei Key Laboratory of Cardiology, Wuhan 430060, China

## Abstract

The current study demonstrates that a disintegrin and metalloproteinase with thrombospondin type 1 motif- (ADAMTS-) 5 is a key extracellular matrix protease and associated with cardiovascular diseases. However, the plasma ADAMTS-5 levels and relevance of coronary artery disease (CAD) remain largely unknown. This study is aimed at examining the relationship between the plasma ADAMTS-5 levels and the severity of coronary stenosis in patients with CAD. In the present study, the expression of ADAMTS-5 was analyzed in coronary artery samples and blood. The results showed that the plasma ADAMTS-5 levels were lower in the CAD group than in the control group. In addition, significantly higher matrix metalloproteinase- (MMP-) 2 and MMP-9 levels were also observed in the patients with CAD, and the ADAMTS-5 levels were negatively correlated with the MMP-2 and MMP-9 levels. Spearman's correlation analysis showed that the Gensini score was negatively correlated with the ADAMTS-5 levels but was positively correlated with the MMP-2 and MMP-9 levels. Receiver-operating characteristic (ROC) analysis revealed that ADAMTS-5, MMP-2, and MMP-9 may have a certain diagnostic value in CAD and that the combination of all three metalloproteinases had a higher diagnostic value. The findings provided a better understanding of the role of ADAMTS-5 in the diagnosis of CAD.

## 1. Introduction

Coronary atherosclerotic disease (CAD) is the most common cardiovascular disease and remains a leading cause of morbidity and mortality worldwide. Narrowing of the arterial lumen and rupture of coronary atherosclerotic plaques with or without luminal thrombosis and vasospasm are currently considered to be the main causes of CAD [1–5]. Several pathophysiologic mechanisms are involved in the process of plaque rupture, including coronary atherosclerotic plaque instability, inflammation, and circumferential wall shear stress. The arteries are composed of three layers: the endothelium, the media consist of smooth muscle cells (SMCs) and elastin, and the adventitia. The extracellular matrix (ECM) of the vasculature is primarily produced by cells originally residing in the cardiovascular tissue, namely, endothelial cells (ECs), SMCs, fibroblasts, and cardiomyocytes [6–8]. Additionally, the ECM is also the most abundant component of normal vessels and atherosclerotic plaque, including fibrous caps. Plaque rupture is increased by a weakened fibrous cap, promoted by the loss of function of the vascular smooth muscle cells (VSMCs) and the breakdown of collagen and ECM that may subsequently lead to acute myocardial infarction (AMI) or stroke [9, 10].

The metalloproteinase superfamily comprises several subfamilies, including the matrix metalloproteinase (MMP), a disintegrin and metalloproteinase (ADAM), and ADAM with thrombospondin type 1 motif (ADAMTS) families. These metalloproteinases are capable of degrading the ECM and play an important role in the development and progression of cardiovascular diseases. The ADAMTS proteases are multidomain proteins that are composed of 19 members, which share a similar structural motif and substrate range. Accumulating evidence suggests that ADAMTS are key ECM proteases associated with ECM turnover, which showed a close association with CAD [11–13]. Previous studies have demonstrated that the plasma ADAMTS-7 level was significantly increased in patients with AMI and was positively correlated with ventricular function after AMI [12, 14]. Similarly, low ADAMTS-13 levels were associated with an increased risk of myocardial infarction [13, 15].

ADAMTS-5, known as aggrecanase-2, has the highest aggrecanase activity and contributes to vascular remodeling [16, 17]. Suna et al. reported that the expression level of ADAMTS-5 was markedly decreased in stent-induced vascular injury, associated with the accumulation of proteoglycans, notably aggrecan and versican [16]. In addition, decreased ADAMTS-5 expression was found in atherosclerosis of apolipoprotein E (ApoE) null mice, associated with the accumulation of aggrecan and versican [18]. However, direct reports regarding circulating ADAMTS-5 levels in patients with CAD is still lacking.

In this study, we detected the expression and localization of ADAMTS-5 in humans with CAD. Furthermore, we also tested the plasma levels of ADAMTS-5 and assayed its correlation with CAD as well as its predictive power for the severity of coronary stenosis.

## 2. Methods

### 2.1. Human Coronary Artery Samples

The study was performed at Renmin Hospital of Wuhan University. Atherosclerotic plaque samples were obtained from the right coronary artery (RCA) of patients with CAD (*n* = 6) who underwent heart transplantation surgery. Normal human coronary arteries were collected from healthy donors (*n* = 6) who were declared brain-dead, but which were not suitable for transplantation as a result of noncardiac reasons. The donor had no apparent history of cardiovascular disease, and the coronary arteries were not damaged in car accident and without pathology. The protocol conformed with the Declaration of Helsinki principles and was approved by the Ethics Committee of Renmin Hospital of Wuhan University. Written informed consent was obtained from each patient.

### 2.2. Western Blotting

Total proteins were extracted from the coronary artery in RIPA lysis buffer as described in our previous study [19]. Protein concentrations were determined using a BCA Protein Assay kit (23227, Thermo Fisher Scientific, USA). Total proteins were separated via sodium dodecyl sulphate polyacrylamide gel electrophoresis and then transferred to a polyvinylidene fluoride membrane (IPFL00010, Millipore, USA). Thereafter, the membranes were blocked with 5% skim milk in Tris-buffered saline for 60 min at 37°C. The membranes were incubated with anti-ADAMTS-5 (GTX100332, 1 : 1000 dilution, GeneTex, USA) and anti-*β*-tubulin (2128S, 1 : 1000 dilution, Cell Signaling Technology, USA) antibodies overnight at 4°C, followed by incubation with secondary antibody at room temperature for 60 min. The blots were detected using a two-color infrared imaging system (Odyssey; LICOR) to quantify protein expression. Specific protein expression levels were normalized to the corresponding *β*-tubulin protein.

### 2.3. Histologic Analysis

The coronary artery tissues were fixed with 4% neutral paraformaldehyde, embedded in paraffin, cut into 4-5 *μ*m slices, and mounted onto slides. Hematoxylin and eosin staining was performed according to a standard process.

Immunohistochemistry staining was also performed as previously described [20]. Briefly, the sections were deparaffinized and blocked with 10% bovine serum albumin, followed by incubation with an anti-ADAMTS-5 antibody (GTX100332, 1 : 100 dilution, GeneTex, USA) for 12 h at 4°C. Thereafter, the sections were incubated with anti-rabbit HRP reagent for 1 h at 37°C and visualized with diaminobenzidine. Finally, the sections were mounted with neutral gums and assessed via light microscopy (Nikon H550L, Tokyo, Japan).

For immunofluorescence staining, the sections were blocked with 10% goat serum for 10 min and subsequently incubated with anti-ADAMTS-5 (GTX100332, 1 : 100 dilution, GeneTex, USA), anti-*α*-smooth muscle cell (*α*-SMA, GTX89701, 1 : 100 dilution, GeneTex, USA), CD31 (ab24590, 1 : 100 dilution, Abcam, USA), and CD68 (ab31630, 1 : 100 dilution, Abcam, USA) overnight at 4°C. Next, the sections were incubated with the appropriate secondary antibodies and DAPI solution at room temperature.

### 2.4. Collection of Human Blood Samples

The study was performed at Renmin Hospital of Wuhan University. Between July 2015 and September 2017, patients who experienced chest pain and underwent coronary angiography (CAG) were recruited. The major exclusion criteria were as follows: (1) age of >80 years, (2) severe hepatic or renal insufficiency, (3) malignancy, (4) condition complicated with infectious diseases, (5) autoimmune diseases, and (6) unclear history of drug use. The 159 remaining patients were divided into the non-CAD (NCAD) (*n* = 36) and CAD groups (*n* = 123) according to clinical symptoms and results of CAG. The CAD group was defined as the presence of luminal diameter equal to or more than 50% in one or more epicardial coronary arteries, and the NCAD group was defined as the normal coronary arteries without any stenosis [21, 22]. Demographic data, body weight, height, biochemical parameters, and medication use were recorded on admission to the hospital.

### 2.5. Blood Sample Measurement

Fasting venous peripheral blood samples were obtained at 6:30–7:30 am and collected into sodium heparin vacutainers following admission of all participants. All plasma samples were centrifuged for 20 min at 4000×g, and the plasma was collected. Thereafter, all samples were stored at −80°C for further analyses.

All the samples were thawed, and the plasma ADAMTS-5 (DY2198-05, R&D Systems, USA), MMP-2 (DY902, R&D Systems, USA), and MMP-9 (DMP900, R&D Systems, USA) levels were measured using the commercially available enzyme-linked immune sorbent assay (ELISA) kits according to the manufacturer's instructions.

### 2.6. Gensini Score

The degree of coronary stenosis was estimated using the Gensini score based on the results of CAG [23]. The method was as follows: ≤25% narrowing was assigned scores of 1; 26-50% narrowing, scores of 2; 51-75% narrowing, scores of 4; 76-90% narrowing, scores of 8; 91-99% narrowing, scores of 16; and total occlusion, scores of 32. For localization, the left main coronary artery was assigned a score of 5; proximal left anterior descending (LAD) and left circumflex (LCX), a score of 2.5; midsegment of the LAD, a score of 1.5; and RCA, distal segment of the LAD, and midsegment of the LCX, a score of 1. Finally, the Gensini scores of the patients were calculated via summation of each coronary stenosis.

### 2.7. Statistical Analysis

The plasma cytokine concentrations and clinical characteristics were presented as median values (with interquartile ranges) and compared using the Mann-Whitney *U* tests. Spearman's correlation was used to calculate the correlations between the plasma ADAMTS-5, MMP-2, and MMP-9 levels and the Gensini score. The diagnostic sensitivity and specificity for plasma cytokines in diagnosing CAD were calculated from the receiver-operating characteristic (ROC) curves, using the subjects without CAD as negative controls. For the establishment of the model with the combination of all three cytokines, the probability value was obtained from logistic regression analysis and then used as a new indicator for the diagnosis of CAD based on ROC curve analysis. The data from western blotting were expressed as means ± SDs and compared using Student's *t*-test. A *P* value of <0.05 was considered to indicate statistical significance. Statistical analyses were performed using SPSS 21.0 and GraphPad Prism7.

## 3. Results

### 3.1. Basic Clinical Characteristics of Patients Who Provided Coronary Tissue Samples

Among the patients who provided coronary tissue, the levels of C-reactive protein (CRP) were higher in the patients with CAD. No differences were found between normal donors and patients with CAD for other clinical characteristics, including gender, age, smoking, glucose (Glu), total cholesterol (TC), triglycerides (TG), low-density lipoprotein cholesterol (LDL-C), high-density lipoprotein cholesterol (HDL-C), systolic blood pressure (SBP), and diastolic blood pressure (DBP). The clinical data for all patients are listed in Table 1.

### 3.2. Expression of ADAMTS-5 in Human Atherosclerotic Plaque Tissue

**Figure 1:**
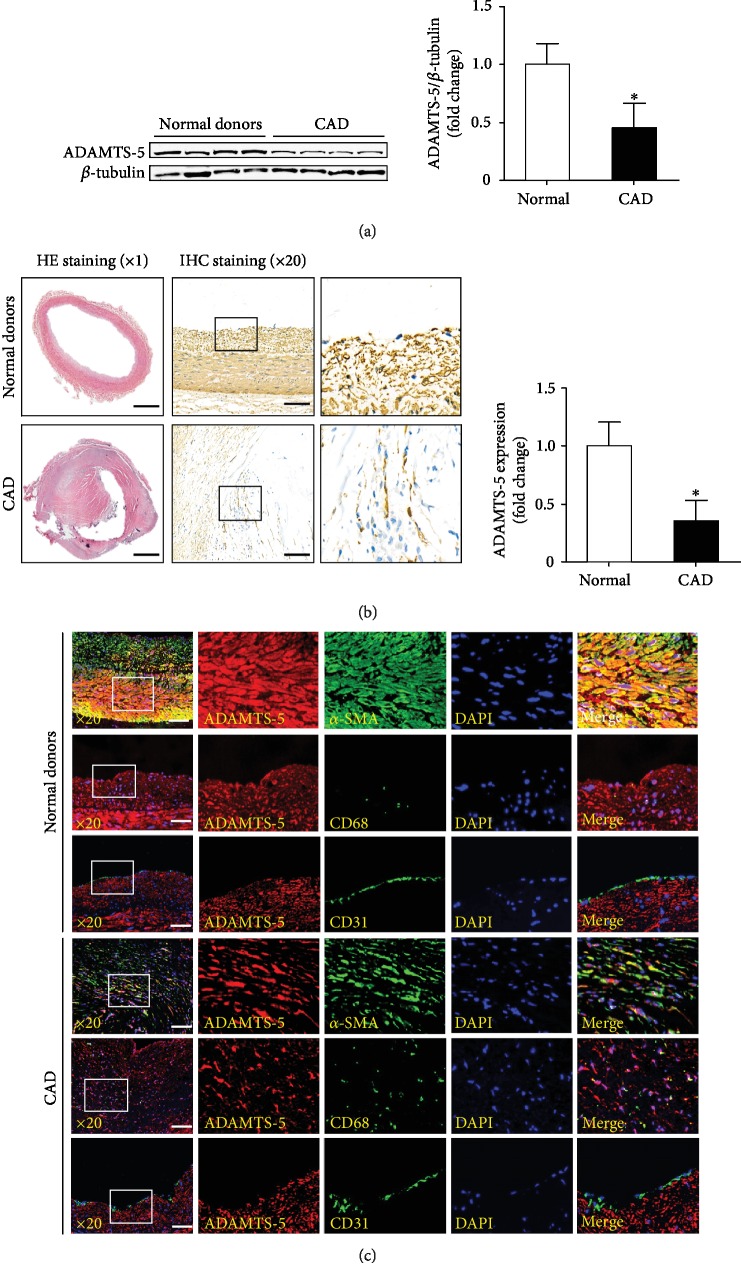
The ADAMTS-5 expression in human atheromatous plaques. (a) Western blot analysis of ADAMTS-5 in atheromatous plaques from normal donors and patients with coronary heart disease (CAD) (*n* = 6/group). ^∗^*P* < 0.05 versus donors. (b) The ADAMTS-5 expression in these two groups were measured by immunohistochemistry staining (*n* = 6/group, scale bar, 1000 *μ*m for the left set of panels and 100 *μ*m for the right panels). (c) The source of ADAMTS-5 was detected by double immunofluorescence staining (*n* = 6/group, scale bar, 100 *μ*m).

To investigate the potential role of ADAMTS-5 in the progression of CAD, we examined whether ADAMTS-5 expression levels were altered in atheromatous plaques. The western blotting and immunohistochemistry results showed that the expression level of ADAMTS-5 significantly decreased in the coronary arteries of the patients with CAD (Figures 1(a) and 1(b)). To further investigate the source of ADAMTS-5 in coronary arteries of the patients with CAD, double-immunofluorescence staining was performed. The results showed that ADAMTS-5 was expressed in VSMCs and macrophages, while VSMCs were the major source of ADAMTS-5 in human CAD atherosclerotic plaques (Figure 1(c)).

### 3.3. Baseline Characteristics of Patients Who Provided Plasma Samples

Baseline clinical characteristics of the two groups are presented in Table 2. There were no significant differences in mean age, gender, smoking, drinking, hypertension, hyperlipidemia, diabetes, Glu, TG, TC, HDL-C, and LDL-C. The incidence rate of the Gensini score and CRP was higher in the CAD group when compared with the NCAD group. Baseline characteristics of the patients are shown in Table 2.

### 3.4. Plasma Cytokine Concentrations in CAD Patients

**Figure 2:**
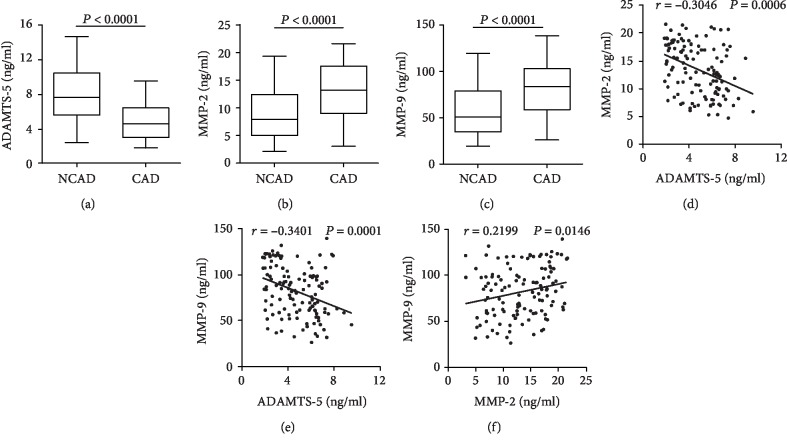
Plasma cytokine levels in each group. The plasma levels of ADAMTS-5 (a), MMP-2 (b), and MMP-9 (c) in NCAD (*n* = 36) and CAD (*n* = 123) groups were measured by ELISA. Spearman's correlation between the plasma levels of MMP-2 (d), MMP-9 (e), and ADAMTS-5 in CAD patients (*n* = 123). (f) Correlation between MMP-2 levels and MMP-9 levels (*n* = 123).

**Figure 3:**
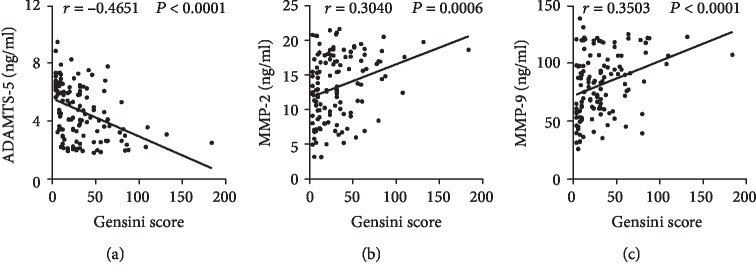
Correlation between plasma cytokine levels and Gensini score. Spearman's correlations between the plasma ADAMTS-5 (a), MMP-2 (b), and MMP-9 levels (c) and the Gensini score in the patients with CAD (*n* = 123).

**Figure 4:**
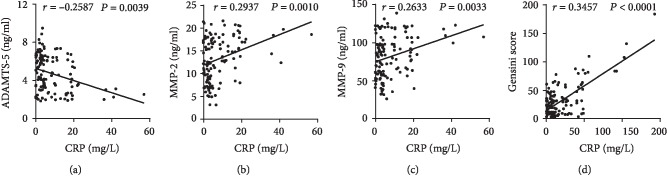
Correlation analysis of ADAMTS-5 (a), MMP-2 (b), and MMP-9 levels (c) and the Gensini score (d) with circulating CRP levels in the patients with CAD (*n* = 123).

Plasma levels of ADAMTS-5, MMP-2, and MMP-9 of each patient were detected by ELISA. The plasma ADAMTS-5 levels were lower in the CAD group than in the control group. The plasma MMP-2 and MMP-9 levels, by contrast, were higher in patients with CAD (Figures 2(a)–2(c)). Spearman's correlation analysis reported that the plasma ADAMTS-5 levels were negatively correlated with the MMP-2 levels (*r* = −0.3042, *P* = 0.0006) and MMP-9 levels (*r* = −0.3399, *P* = 0.0001) (Figures 2(c) and 2(d)), whereas the MMP-2 levels were positively correlated with MMP-9 levels (*r* = 0.2201, *P* = 0.0144) (Figures 2(e) and 2(f)). To analyze whether the plasma cytokine levels were associated with the severity of CAD, the correlations among Gensini score, ADAMTS-5, MMP-2, and MMP-9 levels in CAD patients were assessed. Spearman's correlation analysis showed that the Gensini score was negatively correlated with the ADAMTS-5 levels (*r* = −0.4663, *P* < 0.0001) (Figure 3(a)) but was positively correlated with the MMP-2 (*r* = 0.3135, *P* = 0.0004) and MMP-9 (*r* = 0.3504, *P* < 0.0001) levels (Figures 3(b) and 3(c)). In addition, the circulating CRP were negatively correlated with the ADAMTS-5 levels (*r* = −0.2587, *P* = 0.0039) (Figure 4(a)), whereas positively correlated with MMP-2 levels (*r* = 0.2937, *P* = 0.0010) and MMP-9 levels (*r* = 0.2633, *P* = 0.0033) and the Gensini score (*r* = 0.3457, *P* < 0.0001) (Figures 4(b)–4(d)). The plasma ADAMTS-5, MMP-2, and MMP-9 levels in each group are listed in Table 2.

### 3.5. Simple Linear Regression Analysis and Binary Linear Regression Analysis

To determine the independent predictors of the presence of CAD, simple linear regression analyses and subsequent binary linear regression analyses were performed. Simple linear regression analyses showed that ADAMTS-5, MMP-2, MMP-9, and CRP levels exhibited a trend towards an association with the presence of CAD, whereas gender, age, smoking, SBP, DBP, Glu, TC, TG, HDL-C, and LDL-C showed no obvious trend towards this association. Further, binary linear regression analyses were performed. The results demonstrated that ADAMTS-5 was independently associated with the presence of CAD (as shown in Table 3).

### 3.6. ROC Curves of ADAMTS-5

**Figure 5:**
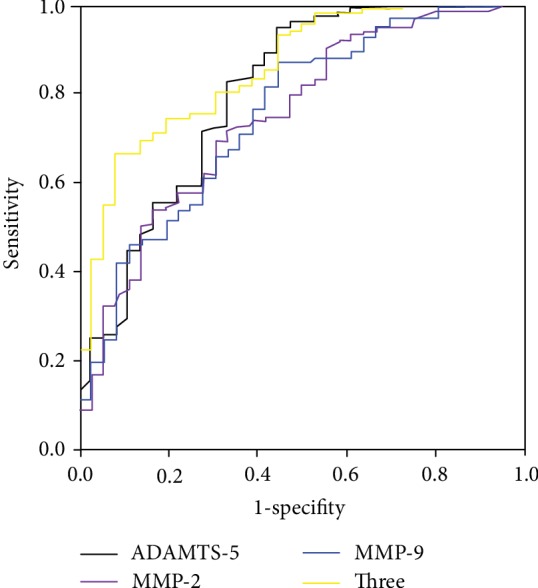
The ROC curve of ADAMTS-5, MMP-2, MMP-9, and combining the three cytokines for the diagnostic value of CAD.

To assess the diagnostic accuracy of ADAMTS-5 in the detection of CAD, we performed ROC curve analysis and found that the area under the curve of ADAMTS-5 was 0.801, which was as high as that of MMP-2 (AUC = 0.748) and MMP-9 (AUC = 0.752) (Figure 5). Furthermore, combination of all three cytokines had a higher area under the curve value (AUC = 0.856) (Figure 5), indicating that combination of all three cytokines has a better capability to predict CAD than when either of the parameters is used alone.

## 4. Discussion

In this study, we found that the expression level of ADAMTS-5 was reduced in the coronary arteries of the patients with CAD, and the VSMCs were the major source of ADAMTS-5 in human CAD atherosclerotic plaques. Furthermore, the results showed that the plasma ADAMTS-5 levels also significantly decreased, whereas the plasma MMP-2 and MMP-9 levels increased in the patients with CAD. Meanwhile, the levels of ADAMTS-5 were negatively correlated with the levels of CAD-associated cytokines, such as MMP-2 and MMP-9. More importantly, ROC analysis showed that reduced ADAMTS-5 levels in patients may have a diagnostic value for CAD.

The ECM, known as matrix or stroma, is an essential constituent of the vessel wall and plays a pivotal role in maintaining the structural integrity of the vascular network [24]. ECM remodeling has been reported to be a critical modulator for the pathogenesis of several cardiovascular conditions, including atherosclerosis, restenosis, and heart failure [25–27]. The ECM not only plays an important role in plaque mass by regulating deposition of proteoglycans and collagen but also governs lipid uptake and accumulation of inflammatory cells within the plaque. It is generally accepted that disturbance of the production and degradation of the ECM could result in destabilizing changes in the plaque tissue and thereby increased susceptibility to AMI [28, 29].

ADAMTS-5 is a member of the evolutionary conserved proteoglycanase clade of the ADAMTS superfamily. A previous study has demonstrated that deficiency of ADAMTS-5 contributed to a marked reduction of versikine and resulted in aortic ECM remodeling in angiotensin II-induced thoracic aortic aneurysm [17]. Ozkaramanli et al. reported that serum ADAMTS-5 concentrations were lower in CAD patients with concomitant peripheral artery disease (PAD) when compared with patients with CAD only [30]. Meanwhile, ADAMTS-5 concentrations were a significant predictor of multiple atherosclerotic involvement (CAD+PAD) and had good diagnostic performances to discriminate multiple manifestations of atherosclerosis. This research showed that ADAMTA-5 may be termed as a potential marker for detection of atherosclerotic involvement at multiple vascular territories. In our study, we aimed to detect the expression of ADAMTS-5 in humans with CAD and assayed its predictive power for the severity of coronary stenosis. The results showed that the ADAMTS-5 expression significantly decreased both in coronary arteries and blood samples in CAD patients. Spearman's correlation analysis also showed that the plasma ADAMTS-5 levels were negatively correlated with the Gensini score and CRP. In addition, binary linear regression analyses showed that ADAMTS-5 was independently associated with the presence of CAD. The results showed that ADAMTS-5 might participate in the onset of CAD and serve as a predictor of CAD.

Components of the ECM, including proteoglycans and collagen, have been identified in atherosclerotic plaques and are synthesized by the VSMCs [31, 32]. It has been demonstrated that the VSMCs play a fundamental role in the pathogenesis of vascular lesions, including lipid retention, ECM production, and plaque integrity maintenance [33, 34]. Previous studies have reported that ADAMTS-5 and proteoglycans were expressed in human coronary arterial VSMCs [16]. Consistent with previous reports, double-immunofluorescence staining indicated that the VSMCs were the major source of ADAMTS-5 in human CAD atherosclerotic plaques. These data also demonstrated that ADAMTS-5 was released from the VSMCs in the arterial wall and ECM turnover was the possible mechanism.

Similar to the ADAMTS family, MMPs are a family of ECM-degrading enzymes and appear to be more active in unstable plaque lesions. Kuzuya et al. reported that MMP-2 deficiency was associated with a decreased incidence of atherosclerotic plaque lesions and accumulation of VSMCs in the ApoE-null mouse [35]. In addition, MMP-9 deficiency in ApoE-null mice also reduced the intimal plaque length, and infiltration of macrophages and migration of the VSMCs [36]. Importantly, accumulating evidence has demonstrated that elevated circulating levels of MMP-2 and MMP-9 were a biomarker for CAD [37–39]. Consistent with previous reports, our results showed that circulating levels of MMP-2 and MMP-9 were remarkably higher in the CAD group than in the control group. In addition, the ROC analysis showed that these three cytokines may have a certain diagnostic value in CAD and that the combination of all three cytokines had a higher diagnostic value. Based on the findings of this analysis, a possible explanation was that decreased ADAMTS-5 levels and increased MMP-2 and MMP-9 levels accelerate ECM remodeling, leading to degradation of the fibrous cap of the vulnerable atherosclerotic plaque and acceleration of plaque rupture.

There are some limitations in our research. First, both the blood sample and coronary artery sample sizes are small, and the patients were not followed up to assess long-term mortality or morbidity. Second, we did not measure the plasma ADAMTS-5 levels in the healthy controls (no chest pain). In conclusion, ADAMTS-5 shows a potential as a novel biomarker for CAD.

## Figures and Tables

**Table 1:** Clinical characteristics in patients who provide coronary tissue samples.

Characteristics	NCAD (*n* = 6)	CAD (*n* = 6)	*P*
Gender (M/F)	3/3	5/1	0.546
Age (years)	57 (46.5, 62)	58 (51.5, 66)	0.485
Smoking (*n*, %)	2 (33.3%)	4 (75%)	0.568
Glu (mmol/L)	5.30 (4.95, 5.75)	5.60 (5.10, 6.35)	0.394
TG (mmol/L)	1.41 (1.04, 1.81)	1.46 (1.32, 1.98)	0.485
TC (mmol/L)	3.35 (2.58, 3.66)	3.87 (3.31, 4.93)	0.240
HDL-C (mmol/L)	0.78 (0.71, 1.18)	0.80 (0.72, 0.99)	0.818
LDL-C (mmol/L)	1.72 (1.29, 1.91)	2.09 (1.27, 3.00)	0.290
SBP (mmHg)	120 (104, 131)	125 (108, 128)	0.589
DBP (mmHg)	72 (64, 85)	68 (62, 77)	0.485
CRP (mg/L)	0.66 (0.49, 0.93)	3.06 (0.77, 8.70)	0.026

Data are expressed as median (interquartile range) or the number (percentage) for category variables. Glu: fasting glucose; TG: total triglycerides; TC: total cholesterol; HDL-C: high-density lipoprotein cholesterol; LDL-C: low-density lipoprotein cholesterol; SBP: systolic blood pressure; DBP: diastolic blood pressure; CRP: C-reactive protein.

**Table 2:** Information of clinical characteristics and laboratory data in the NCAD group and the CAD group.

Characteristics	NCAD (*n* = 36)	CAD (*n* = 123)	*P*
Gender (M/F)	20/16	78/45	0.438
Age (years)	63 (51, 74)	64 (57, 73)	0.465
Smoking (*n*, %)	6 (16.7%)	38 (30.9%)	0.137
Drinking (*n*, %)	2 (5.56%)	22 (17.9%)	0.110
Hypertension (*n*, %)	25 (69.4%)	95 (77.2%)	0.380
Hyperlipidemia (*n*, %)	14 (38.9%)	39 (31.7%)	0.428
Diabetes (*n*, %)	7 (19.4%)	39 (31.7%)	0.210
Glu (mmol/L)	5.33 (4.84, 5.68)	5.53 (4.93, 6.45)	0.119
SBP (mmHg)	147 (139, 155)	144 (127, 158)	0.627
DBP (mmHg)	80 (74, 93)	82 (74, 90)	0.818
HDL-C (mmol/L)	1.06 (0.87, 1.28)	1.00 (0.83, 1.22)	0.548
LDL-C (mmol/L)	2.20 (1.89, 2.54)	2.03 (1.50, 2.58)	0.174
TC (mmol/L)	4.16 (3.65, 5.04)	4.10 (3.34, 4.83)	0.338
TG (mmol/L)	1.38 (1.18, 1.82)	1.45 (0.97, 2.23)	0.951
CRP (mg/L)	0.94 (0.43, 2.06)	4.04 (1.78, 10.4)	<0.001
ADAMTS-5 (ng/mL)	7.62 (5.60, 10.5)	4.64 (3.02, 6.41)	<0.001
MMP-2 (ng/mL)	7.95 (5.06, 12.4)	13.3 (9.09, 17.6)	<0.001
MMP-9 (ng/mL)	50.1 (34.8, 78.9)	83.4 (59.0, 103.4)	<0.001
Gensini score	—	24 (10, 46)	—
Medications, *n* (%)			
Aspirin	13 (36.1%)	62 (50.4%)	0.184
Statin	8 (22.2%)	47 (38.2%)	0.110
ACEI/ARB	17 (47.2%)	66 (53.6%)	0.571
CCB	11 (30.5%)	52 (42.3%)	0.247
*β*-Receptor blockers	6 (16.7%)	35 (28.5%)	0.196
Diuretic	3 (8.33%)	7 (5.69%)	0.696
Insulin	4 (11.1%)	22 (17.8%)	0.446
Oral hypoglycemics	2 (5.56%)	17 (13.8%)	0.248

Data are expressed as median (interquartile range) or the number (percentage) for category variables. Glu: fasting glucose; SBP: systolic blood pressure; DBP: diastolic blood pressure; HDL-C: high-density lipoprotein cholesterol; LDL-C: low-density lipoprotein cholesterol; TC: total cholesterol; TG: total triglycerides; CRP: C-reactive protein; ADAMTS-5: a disintegrin and metalloproteinase with thrombospondin type 1 motif-5; MMP-2: matrix metalloproteinase-2; MMP-9: matrix metalloproteinase-9; ACEI: angiotensin-converting enzyme inhibitor; ARB: angiotensin receptor blocker; CCB: calcium channel blockers.

**Table 3:** Association between cytokines, clinical characteristics, and the presence of acute CAD was assessed by simple linear regression analysis and subsequent binary linear regression analysis.

Variables	Simple linear			Binary linear		
	*β*	95% CI	*P* value	*β*	95% CI	*P* value
ADAMTS-5	-0.518	-0.653 to -0.383	<0.001	-0.352	-0.081 to -0.032	<0.001
MMP-2	0.361	0.214 to 0.508	<0.001	0.147	0.000 to 0.024	0.045
MMP-9	0.365	0.219 to 0.512	<0.001	0.163	0.000 to 0.004	0.028
CRP	0.303	0.153 to 0.454	<0.001	0.144	0.000 to 0.014	0.041
Gender	-0.068	-0.225 to 0.090	0.397			
Age	0.123	-0.034 to 0.279	0.124			
Smoking	-0.133	-0.289 to 0.023	0.094			
SBP	-0.030	-0.187 to 0.128	0.712			
DBP	0.013	-0.145 to 0.171	0.871			
Glu	0.010	-0.100 to 0.121	0.855			
TC	-0.071	-0.228 to 0.086	0.375			
TG	0.024	-0.134 to 0.181	0.766			
HDL-C	-0.095	-0.252 to 0.061	0.231			
LDL-C	-0.082	-0.239 to 0.075	0.304			

ADAMTS-5: a disintegrin and metalloproteinase with thrombospondin type 1 motif-5; MMP-2: matrix metalloproteinase-2; MMP-9: matrix metalloproteinase-9; CRP: C-reactive protein; SBP: systolic blood pressure; DBP: diastolic blood pressure; Glu: fasting glucose; TC: total cholesterol; TG: total triglycerides; HDL-C: high-density lipoprotein cholesterol; LDL-C: low-density lipoprotein cholesterol.

## Data Availability

Data and material related to this manuscript are available from the corresponding authors on reasonable request.

## References

[1] Falk E., Nakano M., Bentzon J. F., Finn A. V., Virmani R. (2013). Update on acute coronary syndromes: the pathologists' view. *European Heart Journal*.

[2] Libby P., Theroux P. (2005). Pathophysiology of coronary artery disease. *Circulation*.

[3] Kishimoto Y., Ibe S., Saita E. (2018). Plasma heme oxygenase-1 levels in patients with coronary and peripheral artery diseases. *Disease Markers*.

[4] Di Ye Z. W., Ye J., Wang M. (2019). Interleukin-5 levels are decreased in the plasma of coronary artery disease patients and inhibit Th1 and Th17 differentiation in vitro. *Revista Española de Cardiología (English Edition)*.

[5] Bom M. J., Levin E., Driessen R. S. (2019). Predictive value of targeted proteomics for coronary plaque morphology in patients with suspected coronary artery disease. *eBioMedicine*.

[6] Frantz C., Stewart K. M., Weaver V. M. (2010). The extracellular matrix at a glance. *Journal of Cell Science*.

[7] Xu J., Shi G. P. (2014). Vascular wall extracellular matrix proteins and vascular diseases. *Biochimica et Biophysica Acta (BBA)-Molecular Basis of Disease*.

[8] Wagenseil J. E., Mecham R. P. (2009). Vascular extracellular matrix and arterial mechanics. *Physiological Reviews*.

[9] Viola M., Karousou E., D'Angelo M. L. (2016). Extracellular matrix in atherosclerosis: hyaluronan and proteoglycans insights. *Current Medicinal Chemistry*.

[10] Fernandez-Hernando C., Jozsef L., Jenkins D., Di Lorenzo A., Sessa W. C. (2009). Absence of Akt 1 reduces vascular smooth muscle cell migration and survival and induces features of plaque vulnerability and cardiac dysfunction during atherosclerosis. *Arteriosclerosis, Thrombosis, and Vascular Biology*.

[11] Salter R. C., Ashlin T. G., Kwan A. P. L., Ramji D. P. (2010). ADAMTS proteases: key roles in atherosclerosis?. *Journal of Molecular Medicine (Berlin, Germany)*.

[12] Wu W., Zhou Y., Li Y. (2015). Association between plasma ADAMTS-7 levels and ventricular remodeling in patients with acute myocardial infarction. *European Journal of Medical Research*.

[13] Maino A., Siegerink B., Lotta L. A. (2015). Plasma ADAMTS-13 levels and the risk of myocardial infarction: an individual patient data meta-analysis. *Journal of Thrombosis and Haemostasis*.

[14] Wu W., Wang H., Yu C. (2016). Association of ADAMTS-7 levels with cardiac function in a rat model of acute myocardial infarction. *Cellular Physiology and Biochemistry*.

[15] Andersson H. M., Siegerink B., Luken B. M. (2012). High VWF, low ADAMTS13, and oral contraceptives increase the risk of ischemic stroke and myocardial infarction in young women. *Blood*.

[16] Suna G., Wojakowski W., Lynch M. (2018). Extracellular matrix proteomics reveals interplay of aggrecan and aggrecanases in vascular remodeling of stented coronary arteries. *Circulation*.

[17] Fava M., Barallobre-Barreiro J., Mayr U. (2018). Role of ADAMTS-5 in aortic dilatation and extracellular matrix remodeling. *Arteriosclerosis, Thrombosis, and Vascular Biology*.

[18] Didangelos A., Mayr U., Monaco C., Mayr M. (2012). Novel role of ADAMTS-5 protein in proteoglycan turnover and lipoprotein retention in atherosclerosis. *The Journal of Biological Chemistry*.

[19] Wang Z., Wang M., Liu J. (2018). Inhibition of TRPA1 attenuates doxorubicin-induced acute cardiotoxicity by suppressing oxidative stress, the inflammatory response, and endoplasmic reticulum stress. *Oxidative Medicine and Cellular Longevity*.

[20] Wang Z., Xu Y., Wang M. (2018). TRPA1 inhibition ameliorates pressure overload-induced cardiac hypertrophy and fibrosis in mice. *eBioMedicine*.

[21] Li C., Chen J. W., Liu Z. H. (2018). CTRP5 promotes transcytosis and oxidative modification of low-density lipoprotein and the development of atherosclerosis. *Atherosclerosis*.

[22] Gao S., Deng Y., Wu J. (2019). Eosinophils count in peripheral circulation is associated with coronary artery disease. *Atherosclerosis*.

[23] Gensini G. G. (1983). A more meaningful scoring system for determining the severity of coronary heart disease. *The American Journal of Cardiology*.

[24] Mouw J. K., Ou G., Weaver V. M. (2014). Extracellular matrix assembly: a multiscale deconstruction. *Nature Reviews. Molecular Cell Biology*.

[25] Chung I. M., Gold H. K., Schwartz S. M., Ikari Y., Reidy M. A., Wight T. N. (2002). Enhanced extracellular matrix accumulation in restenosis of coronary arteries after stent deployment. *Journal of the American College of Cardiology*.

[26] Farb A., Kolodgie F. D., Hwang J. Y. (2004). Extracellular matrix changes in stented human coronary arteries. *Circulation*.

[27] Lynch M., Barallobre-Barreiro J., Jahangiri M., Mayr M. (2016). Vascular proteomics in metabolic and cardiovascular diseases. *Journal of Internal Medicine*.

[28] Chistiakov D. A., Sobenin I. A., Orekhov A. N. (2013). Vascular extracellular matrix in atherosclerosis. *Cardiology in Review*.

[29] Schwanekamp J. A., Lorts A., Vagnozzi R. J., Vanhoutte D., Molkentin J. D. (2016). Deletion of periostin protects against atherosclerosis in mice by altering inflammation and extracellular matrix remodeling. *Arteriosclerosis, Thrombosis, and Vascular Biology*.

[30] Ozkaramanli G. D., Guzel S., Akyuz A., Alpsoy S., Guler N. (2018). The role of novel cytokines in inflammation: defining peripheral artery disease among patients with coronary artery disease. *Vascular Medicine*.

[31] Wight T. N., Merrilees M. J. (2004). Proteoglycans in atherosclerosis and restenosis: key roles for versican. *Circulation Research*.

[32] Wight T. N. (2008). Arterial remodeling in vascular disease: a key role for hyaluronan and versican. *Frontiers in Bioscience*.

[33] Bennett M. R., Sinha S., Owens G. K. (2016). Vascular smooth muscle cells in atherosclerosis. *Circulation Research*.

[34] Grootaert M., Moulis M., Roth L. (2018). Vascular smooth muscle cell death, autophagy and senescence in atherosclerosis. *Cardiovascular Research*.

[35] Kuzuya M., Nakamura K., Sasaki T., Cheng X. W., Itohara S., Iguchi A. (2006). Effect of MMP-2 deficiency on atherosclerotic lesion formation in ApoE-Deficient mice. *Arteriosclerosis, Thrombosis, and Vascular Biology*.

[36] Choi E. T., Collins E. T., Marine L. A. (2005). Matrix metalloproteinase-9 modulation by resident arterial cells is responsible for injury-induced accelerated atherosclerotic plaque development in apolipoprotein E-deficient mice. *Arteriosclerosis, Thrombosis, and Vascular Biology*.

[37] Hlatky M. A., Ashley E., Quertermous T. (2007). Matrix metalloproteinase circulating levels, genetic polymorphisms, and susceptibility to acute myocardial infarction among patients with coronary artery disease. *American Heart Journal*.

[38] Hamed G. M., Fattah M. F. (2015). Clinical relevance of matrix metalloproteinase 9 in patients with acute coronary syndrome. *Clinical and Applied Thrombosis/Hemostasis*.

[39] Dhillon O. S., Khan S. Q., Narayan H. K. (2009). Matrix metalloproteinase-2 predicts mortality in patients with acute coronary syndrome. *Clinical Science (London, England)*.

